# Outcomes of Nonagenarians Commencing Dialysis by Era in the USA

**DOI:** 10.1016/j.ekir.2026.107040

**Published:** 2026-08-19

**Authors:** Donal J. Sexton, Scott Reule, Robert N. Foley

**Affiliations:** 1 Trinity Kidney Centre, Clinical Medicine, School of Medicine, Trinity College Dublin, Ireland; 2 Department of Nephrology, Minneapolis Veterans Administration, Minneapolis, Minnesota, USA

**Keywords:** ageing, dialysis, nonagenarian, United States

## Introduction

As life expectancy improves, the proportion of the population aged ≥90 years is increasing in the US and is associated with a rising prevalence of chronic kidney disease.^1^^,^^2^ Despite being one of the fastest-growing population segments, contemporary data on outcomes of kidney failure in this subgroup are very limited.^2^, ^3^, ^4^, ^5^ Decisions surrounding the initiation of dialysis in those aged ≥90 years are hampered by a lack of specific data on the subsequent typical course experienced, including survival, rates of hospitalization, and dialysis withdrawal. To address some of these questions, we set out to characterize temporal trends in outcomes among nonagenarians with kidney failure in the US (Supplementary Methods).

## Results

A total of 21,530 United States Renal Data System participants aged ≥90 years were included. Frequencies of study participants by era were: 2006–2009 (*n* = 5015), 2010–2013 (*n* = 5518), 2014–2017 (*n* = 5505) and 2018–2021 (*n* = 5492). Of the entire cohort, 42.89% were female, 76.77% were White, 14.82% were African American, 8.06% were Hispanic, and 72.63% were from the Northeast region; 55.36% had their primary kidney disease attributed to hypertension, and 30.8% had diabetes. 92.78% had Medicare coverage, 84.1% had a dialysis catheter as vascular access, 11.97% had an arteriovenous fistula or graft for access, and 3.94% were on peritoneal dialysis. A total of 18.64% were institutionalized, 26.5% required assistance with activities of daily living, and 8.82% were unable to transfer.

### Mortality

Death occurred in 91.5% of the study population over a mean follow-up of 1.4 years (Table 1). Adjusted risk of death rate fell over successive eras and was associated with sex (lower in females); race (lower risk in African American and other races compared with White race); primary cause of end-stage kidney disease (lower risk with hypertension, glomerulonephritis, and cystic disease, and higher in other cause category, compared with diabetes as primary cause of kidney failure); Medicare coverage (higher risk with Medicare coverage); dialysis modality (lower risk with peritoneal dialysis); estimated glomerular filtration rate < 10 ml/min per 1.73 m^2^ (lower risk with estimated glomerular filtration rate < 10 ml/min per 1.73 m^2^ compared with ≥ 10 ml/min per 1.73 m^2^); cardiac failure (higher risk of death), and malignancy (higher risk of death).

**Table 1:** Adjusted hazard ratios for death (91.5% of 21,191 over an average follow-up of 1.4 years)

Variable	Subgroup	AHR	95% CI	*P*-value
	-			
Era	2006–2009			
	2010–2013	0.90	(0.86, 0.93)	< 0.001
	2014–2017	0.86	(0.83, 0.90)	< 0.001
	2018–2021	0.83	(0.79, 0.87)	< 0.001
Sex	Male			
	Female	0.85	(0.82, 0.88)	< 0.001
	Missing	0.72	(0.40, 1.29)	0.27
Race	White			
	African American/Black	0.81	(0.78, 0.85)	< 0.001
	Other	0.75	(0.70, 0.79)	< 0.001
Hispanic ethnicity	No			
	Yes	0.82	(0.78, 0.87)	< 0.001
Veteran	No		(0.00, 0.00)	
	Yes	0.97	(0.93, 1.01)	0.09
Region	Northeast	0.99	(0.95, 1.04)	0.78
	Midwest	1.03	(0.99, 1.07)	0.21
	South	0.95	(0.91, 0.99)	0.02
	West	1.11	(0.97, 1.27)	0.14
Primary kidney disease	Diabetes			
	Hypertension	0.97	(0.93, 1.02)	0.27
	Other	0.98	(0.93, 1.03)	0.48
eGFR	0–4			
	5–9	1.06	(1.00, 1.12)	0.05
	10–14	1.16	(1.10, 1.23)	< 0.001
	15+	1.23	(1.16, 1.31)	< 0.001
	Missing	2.00	(1.12, 3.58)	0.02
Medicare coverage	No			
	Yes	1.34	(1.26, 1.43)	< 0.001
Nephrologist input	No		(0.00, 0.00)	
	Yes	0.88	(0.86, 0.91)	< 0.001
Dialysis access	AV fistula/graft			
	Hemodialysis catheter	1.30	(1.24, 1.36)	< 0.001
	Peritoneal dialysis	1.28	(1.18, 1.39)	< 0.001
BMI	0–19			
	20–24	0.91	(0.87, 0.95)	< 0.001
	25–30	0.84	(0.80, 0.88)	< 0.001
	30+	0.81	(0.76, 0.85)	< 0.001
	Missing	1.06	(0.87, 1.28)	0.57
Serum albumin (g/dL)	0–3.4			
	3.5+	0.85	(0.82, 0.88)	< 0.001
	Missing	0.98	(0.94, 1.01)	0.21
Hemoglobin (g/dl)	0–8.9			
	9+	1.00	(0.97, 1.03)	0.96
	Missing	1.03	(0.98, 1.08)	0.31
Institutionalized	No			
	Yes	1.23	(1.18, 1.28)	< 0.001
Nonambulatory	No			
	Yes	1.14	(1.07, 1.20)	< 0.001
Assisted activities of daily living	No			
	Yes	1.03	(0.99, 1.07)	0.09
Inability to transfer	No			
	Yes	1.26	(1.18, 1.35)	< 0.001
Diabetes	No			
	Yes	0.97	(0.93, 1.01)	0.13
CHF	No			
	Yes	1.20	(1.17, 1.24)	< 0.001
IHD	No			
	Yes	1.03	(0.99, 1.06)	0.14
CVA/TIA	No			
	Yes	1.05	(1.00, 1.11)	0.03
Amputation	No			
	Yes	0.97	(0.83, 1.13)	0.7
COPD	No			
	Yes	1.11	(1.06, 1.16)	< 0.001
Smoking	No			
	Yes	1.01	(0.85, 1.19)	0.93
Alcohol intake	No			
	Yes	0.73	(0.52, 1.05)	0.09
Malignancy	No			
	Yes	1.14	(1.09, 1.19)	< 0.001

AHR, adjusted hazard ratio; AV, arteriovenous; BMI, body mass index; CHF, congestive heart failure; CI, confidence interval; COPD, chronic obstructive pulmonary disease; CVA, cerebrovascular accident; eGFR, estimated glomerular filtration rate; IHD, ischemic heart disease; TIA, transient ischemic attack.

#### One-year mortality

For mortality, with the analysis time restricted to 1 year after dialysis initiation, adjusted hazard ratios were *(P* < 0.001 throughout): 2006–2009: 1 (Ref), 2010–2013: 0.86 (0.82, 0.91); 2014–2017: 0.81 (0.77, 0.86); and 2018–2021: 0.78 (0.74, 0.83).

### Withdrawal From Dialysis

Withdrawal from dialysis occurred in 15.1% of the study population over follow-up (Table 2). Adjusted associations of dialysis discontinuation comprised era; race; Medicare coverage; estimated glomerular filtration rate < 10 ml/min per 1.73 m^2^; and malignancy.

**Table 2:** Adjusted hazard ratios for dialysis discontinuation (15.1% of 21,191 over an average follow-up of 1.4 years-

Variable	Subgroup	AHR	95% CI	*P-*value
	-			
Era	2006–2009			
	2010–2013	1.10	(1.01, 1.21)	0.03
	2014–2017	1.28	(1.16, 1.40)	< 0.001
	2018–2021	1.23	(1.12, 1.36)	< 0.001
Sex	Male	0	(0.00, 0.00)	
	Female	0.98	(0.91, 1.05)	0.58
	Missing	0.89	(0.12, 6.74)	0.91
Race	White	0	(0.00, 0.00)	
	African American/Black	0.51	(0.46, 0.57)	< 0.001
	Other	0.43	(0.36, 0.51)	< 0.001
Hispanic ethnicity	No	0	(0.00, 0.00)	
	Yes	0.51	(0.44, 0.59)	< 0.001
Veteran	No	0	(0.00, 0.00)	
	Yes	0.97	(0.90, 1.05)	0.45
		0	(0.00, 0.00)	
Region	Northeast	1.65	(1.50, 1.81)	< 0.001
	Midwest	1.66	(1.51, 1.82)	< 0.001
	South	1.21	(1.09, 1.35)	< 0.001
	West	0.80	(0.47, 1.34)	0.39
Primary kidney disease	Diabetes	0	(0.00, 0.00)	
	Hypertension	1.03	(0.92, 1.14)	0.63
	other	1.08	(0.96, 1.21)	0.21
eGFR	0–4	0	(0.00, 0.00)	
	5–9	1.01	(0.89, 1.14)	0.9
	10–14	1.16	(1.02, 1.32)	0.03
	15+	1.17	(1.01, 1.34)	0.03
	Missing	0.76	(0.10, 5.53)	0.79
Medicare coverage	No	0	(0.00, 0.00)	
	Yes	1.33	(1.14, 1.55)	< 0.001
Nephrologist input	No	0	(0.00, 0.00)	
	Yes	1.01	(0.95, 1.08)	0.7
Dialysis access	AV fistula/graft	0	(0.00, 0.00)	
	Hemodialysis catheter	1.17	(1.06, 1.29)	< 0.001
	Peritoneal dialysis	1.08	(0.91, 1.29)	0.39
BMI	0–19	0	(0.00, 0.00)	
	20–24	0.97	(0.88, 1.07)	0.54
	25–30	0.86	(0.78, 0.96)	0.01
	30+	0.89	(0.78, 1.00)	0.06
	Missing	1.15	(0.74, 1.78)	0.55
Serum albumin (g/dl)	0–3.4	0	(0.00, 0.00)	
	3.5+	0.94	(0.86, 1.02)	0.13
	Missing	1.05	(0.97, 1.14)	0.26
Hemoglobin (g/dl)	0–8.9	0	(0.00, 0.00)	
	9+	1.08	(0.99, 1.16)	0.07
	Missing	1.13	(1.01, 1.26)	0.03
Institutionalized	No	0	(0.00, 0.00)	
	Yes	1.30	(1.19, 1.43)	< 0.001
Nonambulatory	No	0	(0.00, 0.00)	
	Yes	1.23	(1.08, 1.39)	< 0.001
Assisted activities of daily living	No	0	(0.00, 0.00)	
	Yes	0.98	(0.90, 1.07)	0.62
Inability to transfer	No	0	(0.00, 0.00)	
	Yes	0.92	(0.79, 1.08)	0.32
Diabetes	No	0	(0.00, 0.00)	
	Yes	0.93	(0.85, 1.02)	0.12
CHF	No	0	(0.00, 0.00)	
	Yes	1	(0.93, 1.07)	0.97
IHD	No	0	(0.00, 0.00)	
	Yes	1.08	(1.00, 1.16)	0.06
CVA/TIA	No	0	(0.00, 0.00)	
	Yes	1	(0.89, 1.11)	0.94
Amputation	No	0	(0.00, 0.00)	
	Yes	1.06	(0.74, 1.52)	0.74
COPD	No	0	(0.00, 0.00)	
	Yes	1	(0.89, 1.11)	0.95
Smoking	No	0	(0.00, 0.00)	
	Yes	1.21	(0.86, 1.69)	0.27
Alcohol intake	No	0	(0.00, 0.00)	
	Yes	0.57	(0.24, 1.37)	0.21
Malignancy	No	0	(0.00, 0.00)	
	Yes	1.18	(1.08, 1.30)	< 0.001

AHR, adjusted hazard ratio; AV, arteriovenous; BMI, body mass index; CHF, congestive heart failure; CI, confidence interval; COPD, chronic obstructive pulmonary disease; CVA, cerebrovascular accident; eGFR, estimated glomerular filtration rate; IHD, ischemic heart disease; TIA, transient ischemic attack.

### Hospitalization

The overall rate of days hospitalized was 53.3 (95% confidence interval, 53.2–53.4) days per year and fell initially in successive era but rose in the most recent eras studied. The overall rate of days in intensive care was 5.1 days per year. Risk of critical care admission varied by era; it was higher in successive eras and then exhibited a plateau in comparison with the reference era of 2006–2009.

## Discussion

In this large, national study of 21,530 nonagenarians initiating dialysis in the US, we identified a significant and progressive improvement in survival over an 18-year period. This finding suggests that advancements in medical care and patient selection may be conferring a mortality benefit in this population. It is plausible that over time, clinicians have become more discerning in recommending dialysis, offering it to healthier, less comorbid individuals who are inherently more likely to survive longer.

An additional and clinically important finding of this study is the substantial burden of care associated with dialysis in this age group. Patients spent an average of 53.3 days per year hospitalized, equivalent to nearly 2 months annually. Hospitalization rates were highest in the most recent era, despite improved survival, which is paradoxical and may possibly reflect that patients are living longer with a higher burden of chronic disease, leading to more frequent hospital admissions.

Our finding of a low overall rate (15.1%) of dialysis withdrawal before death suggests that there may be opportunities to optimize palliative care input as maintenance dialysis was continued till the end of life, however we cannot confirm this.

Few studies have detailed the clinical epidemiology of kidney failure in nonagenarian patients by era. The most comprehensive assessment of dialysis outcomes in the oldest population segments to date was performed in 2007 and has not yet been updated.^3^ Our study assessed nonagenarians as a unique cohort over multiple 3-year periods from 2006 to 2021, which may allow for a more comprehensive assessment of survival.^3^

Our study has limitations, among which are the registry-based and retrospective design, and the inability to compare conservative management in this age group because of the lack of capture of kidney failure cases not treated with dialysis.^6^ Despite its limitations, our study is, to our knowledge, the largest to date on nonagenarian dialysis initiates and may provide estimates for the outcomes of interest for both patients and providers. The trade-off between a trend of improving survival and the possibility of increased inpatient care ought to be acknowledged and the decision to initiate dialysis in this demographic group should be individualized. This study provides vital natural history information for patients, families, and clinicians to engage in meaningful shared decision-making, balancing the potential for extended life with the realities of the treatment burden.^7^

## Disclosure

All authors declare no competing interests.

## Footnotes

Supplementary File (PDF)

**Supplementary Methods.**
