# Supplementary material for: Outcomes of Nonagenarians Commencing Dialysis by Era in the USA
Source: Kidney Int Rep. 2026 Aug 19;11(12):107040. doi: 10.1016/j.ekir.2026.107040 (PMC13720137; doi:10.1016/j.ekir.2026.107040)
Supplement: Supplementary file 1 [file mmc1.pdf]

Supplementary Methods

Population

Among US dialysis patients, the primary objective of this retrospective study was to evaluate the association between era of initiation and survival of patients ≥ 90 years of age between 2006 and 2021 with follow up ending in June 2022 (n = 21,530). Secondary objectives included describing associations with dialysis withdrawal, rates of hospitalization, and admission to the intensive care (n = 19,975).

Data Source

The United States Renal Data System (USRDS) data sources were the Centers for Medicare and Medicaid (CMS) Medical Evidence Report (form CMS-2728), the Medevid95, Medevid05 files, the Patients file, the death files and the hosp files. Form CMS-2728 was used to determine baseline characteristics at Dialysis initiation. Form CMS-2728 is a Federal requirement for all new maintenance Dialysis patients in the US, with corresponding data available in the USRDS Medevid95 and Medevid05 files. Form CMS-2728 changed twice in the past two decades, in 1995 and 2005. For this study, the primary outcome was all cause mortality. Dates of death were obtained from the Patients file; dates for withdrawal of dialysis were obtained from the death files, hospitalization was determined using the "saf.hosp" files. Local VA IRB Approval applied to this study.

Analysis, Variables & Outcomes

Chi^2 analysis was used for comparison of categorical variables, proportional hazards regression was used for time to event comparators of clinical outcomes, and Poisson regression was used to calculate rate ratios, with use of the 2000 - 2002 biennium as

reference. For time to event analysis with all-cause mortality as the outcome of interest follow-up ranged between initiation of dialysis and the first occurrence of death or June 30th, 2022. For time to event analysis with dialysis discontinuation as the outcome of interest follow-up ranged between initiation of dialysis and the first occurrence of dialysis discontinuation, death or June 30th, 2022. SAS 9.4 (Cary, North Carolina) was used for data evaluation. Variables included for adjustment included in each specific model adjustment are listed beneath each table presented.
